# Circulating microRNAs Related to Bone Metabolism in HIV-Associated Bone Loss

**DOI:** 10.3390/biomedicines9040443

**Published:** 2021-04-20

**Authors:** Maria P. Yavropoulou, Artemis Kolynou, Polyzois Makras, Maria Pikilidou, Sideris Nanoudis, Lemonia Skoura, Olga Tsachouridou, Georgios Ntritsos, Alexandros Tzallas, Dimitrios G. Tsalikakis, Olga Tsave, Simeon Metallidis, Dimitrios Chatzidimitriou

**Affiliations:** 1 Endocrinology Unit, The First Department of Propaedeutic and Internal Medicine, Medical School, National and Kapodistrian University of Athens, 11527 Athens, Greece; 2 Laboratory of Medical Research, 251 Hellenic Air Force & VA General Hospital, 11525 Athens, Greece; pmakras@gmail.com (P.M.); tsaveolga@gmail.com (O.T.); 3 Department of Microbiology, AHEPA University Hospital, Medical School, Aristotle University of Thessaloniki, 54636 Thessaloniki, Greece; mollyskoura@gmail.com; 4 First Department of Internal Medicine, AHEPA University Hospital, Medical School, Aristotle University of Thessaloniki, 54636 Thessaloniki, Greece; pikilidou@gmail.com (M.P.); sidnanoudis@yahoo.gr (S.N.); olgat_med@hotmail.com (O.T.); metallidissimeon@yahoo.gr (S.M.); 5 Department of Informatics & Telecommunications, School of Informatics & Telecommunications, University of Ioannina, 47100 Arta, Greece; gntritsos@uoi.gr (G.N.); tzallas@uoi.gr (A.T.); 6 Department of Engineering Informatics and Telecommunications, University of Western Macedonia, 50100 Kozani, Greece; tsalikakis@gmail.com; 7 National AIDS Reference Centre of Northern Greece, Medical School, Aristotle University of Thessaloniki, 54636 Thessaloniki, Greece; dihi@auth.gr

**Keywords:** circulating miRNAs, HIV infection, antiretroviral therapy, osteoporosis, osteoblasts, osteoclasts, bone metabolism

## Abstract

The pathophysiology of human immunodeficiency virus (HIV)-associated bone loss is complex and to date largely unknown. In this study, we investigated serum expression of microRNAS (miRNAs) linked to bone metabolism in HIV-associated bone loss. This was a case-control study. Thirty male individuals with HIV infection (HIV+) and osteoporosis/osteopenia (HIV+/OP+) (cases) and 30 age-matched male HIV+ individuals with normal bone mass (HIV+/OP−) (controls) were included in the analysis. Thirty male individuals matched for age without HIV infection (HIV−), were also included as second controls. The selected panel of miRNAs was as follows: hsa-miRNA-21-5p; hsa-miRNA-23a-3p; hsa-miRNA-24-2-5p; hsa-miRNA-26a-5p; hsa-miRNA-29a-3p; hsa-miRNA-124-3p; hsa-miRNA-33a-5p; and hsa-miRNA-133a-3p. Within the cohort of HIV+ individuals, relative serum expression of miRNA-21-5p and miRNA-23a-3p was significantly lower (*p* < 0.001) while the expression of miRNA-24-2-5p was significantly higher (*p* = 0.030) in HIV+/OP+ compared to HIV+/OP−. Expression of miRNA-21-5p demonstrated a sensitivity of 84.6% and a specificity of 66.7 in distinguishing HIV+/OP+ individuals. Expression of circulating miRNAs related to bone metabolism; miRNA-23a-3p, miRNA-24-2-5p, and miRNA-21-5p is significantly altered in HIV+OP+ individuals, in line with data on other causes of osteoporosis, suggesting a common pattern of circulating miRNAs independent of the underlying cause.

## 1. Introduction

The prevalence of osteopenia and osteoporosis is greatly increased in individuals with human immunodeficiency virus (HIV+) of all ages compared to the general population [1,2], reaching up to 67% and 15%, respectively. Similarly, HIV+ individuals experience a higher low-trauma fracture risk, ranging from 1.6 to 3-fold, compared to the general population, especially after initiation of highly active antiretroviral therapy (HAART) [3,4].

The pathophysiology of HIV-associated bone loss is complex and still not fully understood [5,6]. In addition to traditional osteoporotic risk factors, direct effects of HIV-1 viral proteins and inflammatory cytokines on bone cells as well as the need for long-term HAART are also implicated [6]. As HIV-associated bone loss is associated with increased osteoclastic activity, the impact of the impaired immune system response [7,8] and the effect of increased proinflammatory cytokines in osteoclastogenesis [9] have been thoroughly studied. However, it has been suggested that a direct effect of viral proteins in osteoclasts plays a key role in the increased osteoclastic activity seen in HIV-associated bone loss. A recent experimental study demonstrated the presence of HIV-infection in osteoclasts of HIV-1-infected humanized mice and of human synovial explants exposed to the HIV-1 virus showing that, at the cellular level, the negative factor (NEF) gene viral protein has an essential role in osteoclast number and function [10].

Currently available screening tools that are used for the classification of patients with osteoporosis and increased fracture risk in the general population include the dual X-ray absorptiometry (DXA) scan and the fracture risk assessment tool (FRAX), which latter estimates the 10-year probability of fracture. A recent analysis conducted in Greece demonstrated no significant differences in the cost-effective FRAX^®^-based thresholds between HIV+ individuals and the general population [11], whereas the measurement of bone mineral density (BMD) with DXA alone is likely to underestimate the risk for fragility fractures in this population [12].

MicroRNAs (miRNAs) are small non-coding single-stranded RNA molecules of approximately 22 nucleotides that act at the post-transcriptional level and directly modulate gene expression by repressing or completely inhibiting expression of the messenger RNA (mRNA) of the respective genes [13]. Cytoplasmic miRNAs are released into the circulation by microvesicles, exosomes, or in microparticle-free form [14]. Due to their generally long half-life, they remain stable in the circulation for 5 days or longer [15], and because the “information” that they carry reflects changes at tissue level, miRNAs are considered promising novel diagnostic biomarkers.

With regard to HIV infection, a miRNA panel of four differentially expressed miRNAs (namely miRNA-16-5p, miRNA-20b-5p, miRNA-195-5p, and miRNA-223-3p) was recently identified in HIV+ individuals using a miRNA PCR-array method showing 100% sensitivity and specificity in distinguishing persons with early HIV-1 infection from healthy individuals [16]. A critical finding in this study was the observation that this miRNA panel was able to identify even viral RNA-negative early-stage HIV-1 infection, lending further support to the potential role of circulating miRNAs as novel and promising biomarkers for the detection of early HIV-infection and HIV-induced complications.

In line with these results, data from earlier studies had shown a differential expression of miRNA profile in the serum of HIV+ individuals in association with HIV-induced vascular disease [17], myocardial infarction, and nephropathy [18,19].

Several in vitro and ex vivo studies have shed light on the role of miRNAs in the regulation of cellular functions of bone cells [20]. In osteoporotic patients, tissue expression of certain miRNAs is highly associated with their presence in serum, thus suggesting that circulating levels of these miRNAs may be directly linked to altered bone metabolism [21]. In addition, we [22,23,24,25] and others [21] have identified a specific expression profile of miRNAs linked to bone metabolism in postmenopausal osteoporosis (PMO) and chronic kidney disease-metabolic bone disease (CKD-MBD), although data are currently lacking regarding HIV-associated bone loss.

In order to determine whether HIV-associated bone loss is associated with and can be predicted by certain alterations in the expression of bone-related miRNAs in serum, we investigated the expression profile of circulating miRNAs that are linked to bone metabolism in male HIV+ individuals with osteoporosis.

## 2. Patients and Methods

This was a case-control study conducted in a University hospital setting.

### 2.1. Patient Recruitment

The reference population was adult male HIV+ individuals (who accounted for approximately 80% of HIV+ individuals registered in the hospital’s database) who were followed regularly in the Unit of Infectious Diseases in AHEPA University Hospital, Thessaloniki, Greece. Inclusion criteria were (i) age > 18 years old and (ii) HAART for at least 1 year. Patients that fulfilled the inclusion criteria were recruited during their regular visit in the outpatient clinic of the Unit of Infectious Diseases in AHEPA University Hospital.

Exclusion criteria were as follows: (1) diabetes mellitus; (2) renal deficiency based on creatinine clearance <30 mil/min (Cockroft–Gault calculation); (3) liver disease; (4) bone diseases other than osteoporosis (i.e., Paget’s disease of bone, rheumatoid arthritis, bone metastatic disease, and primary hyperparathyroidism); (5) untreated hypo- or hyperthyroidism; and (6) previous (last 3 years) or current treatment with medication known to affect bone metabolism (e.g., bisphosphonates, glucocorticoids, calcimimetics, etc.).

Thirty male HIV+ who were diagnosed with osteoporosis or osteopenia based on the World Health Organization (WHO) criteria (defined as a T-score of <−2.5 SD for osteoporosis and a T-score between −1 and −2.5 SD for osteopenia) (cases) and 30 male HIV+ with normal bone mass who were age- and HAART-duration-matched with cases (controls) were included in the analysis at a 1:1 ratio. The mean duration of HAART in the study cohort (*n* = 60) was 10 ± 7.0 years (range between 2 and 26 years).

Thirty adult male HIV− and age-matched individuals were recruited from the medical and paramedical personnel of AHEPA University Hospital and included in the analysis as a second control group. The study was approved by the Scientific Review Board of AHEPA University Hospital (protocol number 6645/17). Informed consent was obtained from all participants. All procedures performed were in accordance with the ethical standards of the institutional research committee and with the 1964 Declaration of Helsinki and its later amendments.

### 2.2. Samples Collection and Storage

Following an overnight fast, morning blood samples were obtained from HIV+ and HIV− individuals. Serum was separated and stored at −80 °C until further analysis.

### 2.3. RNA Isolation/RT qPCR

MiRNAs were extracted from 200 μL of serum samples using the miRNeasy Serum/Plasma Kit, according to the manufacturer’s instructions (Qiagen GmbH, Hilden, Germany), as previously described [22,23,24,25]. During the purification process, a synthetic RNA sequence (spike-in control; Caenorhabditis elegans, miRNA-39-3p) was added in the appropriate amount to serum preparations after homogenization with the QIAzol lysis reagent to control for variations in recovery and amplification efficiency between RNA preparations. RNA quality was assessed using the NanoDrop ND-1000 ^®^ (Thermo Fisher Scientific, Wilmington, NC, USA) and the ratio of absorbance readings at 260 nm and 280 nm (A260/A280) in most of the samples was approximately ~2.0. Reverse transcription was performed with the miScript II RT Kit (Qiagen GmbH, Hilden, Germany). Two snoRNAs (SNORD95 and SNORD96A) and one snRNA (RNU6-2) (Table 1) were used to normalize for variability in sample loading and real-time RT-PCR efficiency. Cycling was performed under standardized conditions with QuantiTect^®^ SYBR Green PCR Master Mix on the QIAGEN Rotor-Gene Q (Corbett Rotor-Gene 6000) real-time PCR cycler (Qiagen GmbH, Hilden, Germany)).

#### MiRNA Primer Assays

We searched for changes in the relative expression of miRNA-21-5p, miRNA-23a-3p, miRNA-24-2-5p, miRNA-26a-5p, miRNA-29a-3p, miRNA-124-3p, miRNA-33a-5p, and miRNA -133a-3p (Table 1). The following databases: (1) miRBase [39], (2) DIANA TOOLS [40], (3) PicTar [41], (4) miRDB [42], (5) TargetScanHuman [43], (6) miRGator [44], and (7) miRNA [45] were searched to confirm the biological targets of the selected miRNAs in humans, searching for 8mer, 7mer, and 6mer sites that match the seed region for each miRNA, using conserved sites and the best cumulative scores.

### 2.4. DXA Measurements

Measurements of BMD were performed by DXA (Lunar Prodigy, General Electric, San Francisco, CA, USA) at the lumbar spine (L1–L4) (LS BMD) and at two femoral sites, femoral neck [FN-BMD] and total femur [TH-BMD]. The coefficients of variations were between 2.5% and 3.7% at LS, 2.2% and 3.1% at FN, and 1.5% and 2.7% at TH, as previously described [46]. Osteopenia and osteoporosis were defined according to WHO criteria as BMD between 1 and 2.5 standard deviations (SD) (osteopenia) or 2.5 SD (osteoporosis) below the average value for young healthy individuals (T-score).

Trabecular bone score (TBS) was evaluated in the same regions as those used for LS-BMD (L1–L4) employing TBS iNsight (Version 1.8, Med-Imaps, Pessac, France). TBS was calculated as the mean value of the individual measurements for the L1–L4 vertebrae. Vertebrae excluded for BMD assessment were also excluded for TBS evaluation at the LS. The coefficient of variation for TBS was between 2.2% and 3.5% [46].

Vertebral fracture assessment (VFA) analysis was performed with DXA at the LS and in the same session with BMD measurement in all enrolled participants to detect any morphometric vertebral fractures.

### 2.5. Biochemical Assays

All assays were measured by a second-generation electrochemiluminescence immunoassay on a Cobas e411 automated analyzer (Roche Diagnostics, Mannheim, Germany), according to the manufacturer’s instructions and as previously described [14]. In brief, the measurement range and total analytical imprecisions for the measured bone parameters were as follows: intact parathyroid hormone (PTH), 1.2 to 5000 pg/mL and 4.0%, respectively; C-terminal cross-linking telopeptide of type I collagen (β-CTX), 10 to 6000 ng/L and 3.5%, respectively; total procollagen type 1 N-terminal propeptide (P1NP), 5 to 1200 ng/mL and 4.5%, respectively; and total 25-OH-Vitamin D levels, 3 to 100 ng/mL and 4.7%, respectively.

### 2.6. Data Analysis

Resultant data on mean Ct values for each miRNA were exported and uploaded to the QIAGEN website for analysis (miRNA primer assay data analysis version 3.5, GeneGlobe Data Analysis), where a classic ΔΔCt calculation and a log2 transformation provided normalized fold-difference values for the miRNA targets. Mean Ct values less than 33 were used as the cutoff threshold, as recommended by the software instructions. All analyses were based on fold-change (2^(-Delta Delta Ct)) defined as the normalized gene expression (2^(-Delta Ct)) in cases (HIV+ with low bone mass) divided by the normalized gene expression (2^(-Delta Ct)) in controls (HIV+ with normal bone mass), or between HIV+ and HIV-controls. Fold-change values less than 1 are indicative of down-regulation of gene expression, and fold-change values greater than 1 indicate up-regulation, accordingly. The *p* values were calculated based on a Student’s t-test of the replicate 2^(-Delta Ct) values for each gene in cases and controls.

### 2.7. Statistical Analysis

The Shapiro–Wilk test was used to assess for normality of distributions; we present our results as mean ± SD or mean (range), as applicable. Student’s t-test was used for mean comparison of parametric variables and the Mann–Whitney U-test if otherwise distributed. Analysis of Variance (ANOVA) test was used for mean comparison of parametric variables in multiple groups.

Pearson correlation coefficient or Spearman’s rank correlation coefficient was used for associations between relative serum expression of miRNAs, BMD values, TBS values, type of HAART, HAART duration, and biochemical parameters of calcium metabolism, as applicable. Receiver operating characteristic (ROC) curves were generated to assess the specificity and sensitivity of miRNA serum expression in distinguishing low bone mass in HIV+. All *p* values are two-sided and a value of *p* < 0.05 was considered statistically significant. Statistical analysis was performed using the IBM SPSS Statistics for Windows, Version 26 (IBM SPSS Statistics for Windows, IBM Corporation, Armonk, NY, USA). Figures were created using GraphPad Prism for Windows version 7 (GraphPad Software San Diego, CA, USA).

## 3. Results

### 3.1. Study Population

The anthropometric, clinical characteristics and biochemical measurements of the study cohort are depicted in Table 2. None of the participants had a history of a prior vertebral or non-vertebral fracture. Serum 25-OH-vitamin D levels were lower in HIV+ compared to HIV− individuals (16.5 ± 7.2 vs. 23.9 ± 16 ng/mL *p* = 0.055), with 70% of HIV+ individuals suffering from vitamin D deficiency (defined as 25-OH-vitamin D levels < 20 ng/mL). Serum levels of the measured bone turnover markers, P1NP, and beta-CTX, did not differ significantly between groups, although they tended to be higher in HIV+ compared to HIV− individuals (Table 2).

In HIV+/OP+ group (cases), 23 were diagnosed with osteoporosis and seven with osteopenia. As expected, cases had significantly lower TBS values compared to HIV+/OP− group (controls). Five (16%) of the HIV− individuals were diagnosed with osteoporosis, according to WHO criteria, based on the BMD measurements performed during enrollment in the study. None of the osteoporotic HIV− individuals had clinical and/or laboratory evidence of secondary osteoporosis. All HIV+ individuals were treated with combinations of antiretroviral regimes. Combinations included two nucleoside reverse-transcriptase inhibitors (NRTIs) as a “backbone” along with one non-nucleoside reverse-transcriptase inhibitor (NNRTI), protease inhibitor (PI), or integrase inhibitor (IN). Duration of each HAART category is presented in Table 2.

### 3.2. Differential Expression of the Selected Panel of miRNAs Linked to Bone Metabolism in HIV+ Individuals with Osteoporosis Compared to HIV+ Individuals with Normal Bone Mass

**Figure 1:**
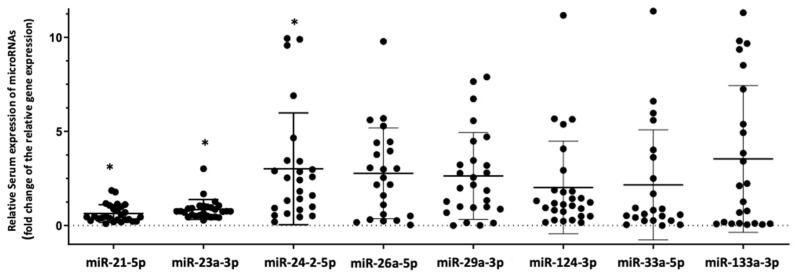
Relative serum expression of the tested miRNAs in HIV+/OP+ (cases) compared to HIV+/OP− (controls). Values are expressed as gene fold changes only in cases *: *p* values < 0.05.

Circulating miRNA expression pattern differed significantly between HIV+/OP+ and HIV+/OP−. Relative serum expression of two miRNAs, namely miRNA-21-5p and miRNA-23a-3p, was significantly lower in HIV+/OP+ compared to HIV+/OP− (fold-change: 0.5, *p* < 0.001 and fold-change: 0.75, *p* = 0.042, respectively), while relative serum expression of miRNA-24-2-5p was significantly higher (fold-change: 2.34, *p* = 0.030) (Figure 1).

**Figure 2:**
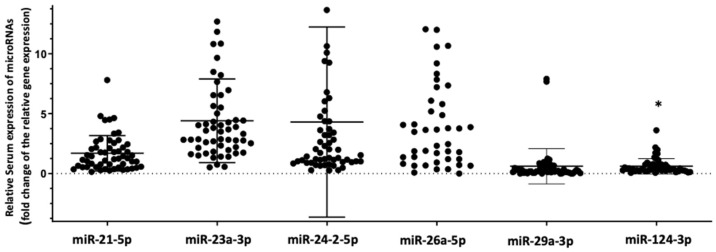
Relative serum expression of the tested miRNAs in HIV+ compared to HIV− individuals. Values are expressed as gene fold changes only in HIV+ individuals *: *p* values < 0.05.

In order to test whether the differences we found in the cohort of HIV+ individuals are mainly due to HIV infection itself and not HIV-associated osteoporosis we also investigated for differences in the relative serum expression of the tested microRNAs between HIV+ (*n* = 60) and HIV− individuals (*n* = 30). In this analysis, only the relative serum expression of miRNA-124-3p was significantly lower (fold-change 0.43, *p* = 0.019) in HIV+ compared to HIV− individuals (Figure 2).

### 3.3. Correlations between HAART, BMD Values, TBS Values, Biochemical Characteristics, and Relative Serum miRNA Expression

Analysis within the cohort of HIV+ individuals showed significant and inverse correlations between BMD values of FN but not LS and the duration of treatment with PIs (*r* = −0.432, *p* = 0.028).

No correlations were observed between the type of HAART combination or the duration of HAART treatment and relative expression of the tested miRNAs in serum. However, in a separate analysis for each HAART drug class, relative expression of miRNA-29a-3p was correlated with the duration of treatment with NRTIs (*r* = 0.393, *p* = 0.043) and, in particular, with TDF (*r* = 0.387, *p* = 0.046), while relative expression of miRNA-24-2-5p was significantly correlated with the duration of NNRTI treatment (*r* = 0.568, *p* = 0.002).

In addition, TBS in the lumbar spine, which estimates bone texture, was positively and significantly correlated with relative expression of miRNA-124-3p in serum (*r* = 0.432, *p* = 0.028) of HIV+ individuals.

**Figure 3:**
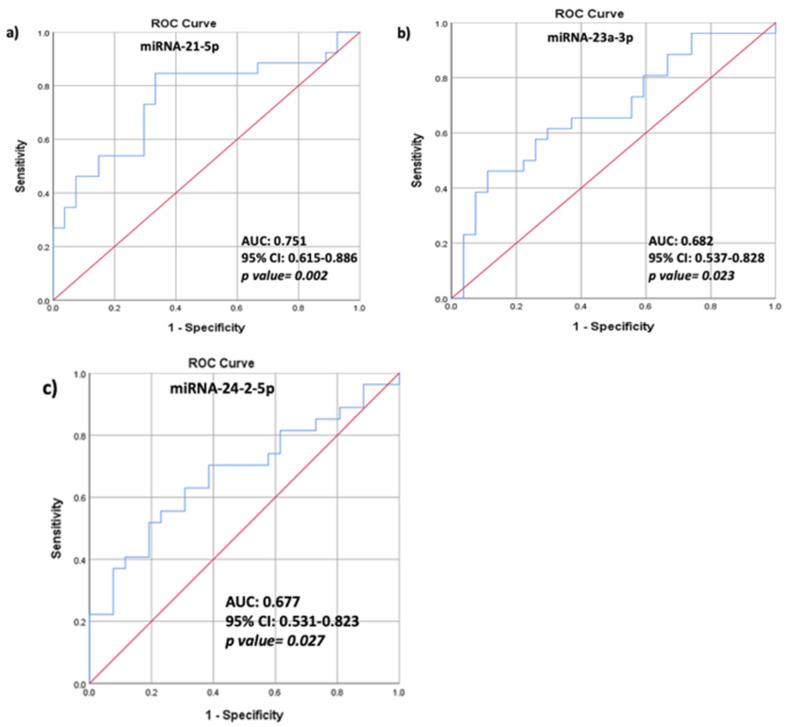
Area under the curve (AUC) of receiver-operating characteristic (ROC) for relative serum expression of (**a**) miRNA-21-5p, (**b**) miRNA-23a-3p, and (**c**) miRNA-24-2-5p and ROC curve in HIV+. AU, 95% confidence interval (95%CI), and nominal *p* values are demonstrated.

ROC analysis was performed to assess the predictive ability of serum miRNA expression in distinguishing HIV+/OP+. The associated area under the curve (AUC) for miRNA-21-5p was 0.751 (95% CI 0.615–0.886; *p* = 0.002), showing a sensitivity of 84.6% and specificity of 66.7% (Figure 3a). The AUC for miRNA–23a-3p was 0.682 (95% CI 0.537–0.828; *p* = 0.023), showing a sensitivity of 65.4% and specificity of 63% (Figure 3b), and the AUC for miRNA-24-2-5p was 0.677 (95% CI 0.531–0.823; *p* = 0.027) showing a sensitivity of 70.4% and specificity of 53.8% (Figure 3c).

## 4. Discussion

In the present study, we show an altered expression pattern of circulating miRNAs that are related to bone metabolism and have been identified in previous studies with PMO [22,23,24] and CKD-MBD [25] in male HIV+ individuals with osteopenia/osteoporosis. In particular, we report significantly lower relative serum expression of miRNA-21-5p and miRNA-23a-3p and higher relative expression of miRNA-24-2-5p in the serum of HIV+/OP+ compared to HIV+/OP−. In addition, we show that serum expression of miRNA-21-5p displays 84.6% sensitivity and 66.7% specificity in distinguishing among male HIV+ those with osteoporosis.

MiRNAs play an important regulatory role in cellular homeostasis in normal conditions, maintaining protein expression within cells. However, they are clearly altered in HIV-1 infected cells, contributing to the pathological decline of cellular function [47]. Their high stability in the circulation and their correlation with molecular mechanisms that occur at the cellular level render miRNA research a very promising field in the quest for novel diagnostic biomarkers. In this context, several studies have identified a panel of circulating miRNAs that are altered during early stages of HIV infection and therefore could enable early diagnosis when expression of HIV viral markers is absent or very low [16,48]. In addition, expression of certain miRNAs in HIV+ has been linked with related complications such as miRNA-222 expression with the development of non-Hodgkin lymphoma [49], and miRNA-32 with HIV− and HAART-related vascular disease [17], while significantly lower expression of miRNA-200 and miRNA-33 was associated with HIV- induced nephropathy [18,19].

In our study, we specifically searched for miRNAs that regulate genes with a key role in bone remodeling.

MiRNA-21-5p is highly expressed in osteoclast precursors and is up-regulated during RANKL-induced osteoclastogenesis [26,27]. In addition, miRNA-21-5p targets *SMAD* and *RUNX2* gene expression, this highlighting its role in osteogenic differentiation as well [49]. On the other hand, miRNA-23a-3p inhibits RUNX2 translation [29], which is the main osteoblastogenic transcription factor. In vivo studies have demonstrated that suppression of miRNA-23a-3p increases osteoblast proliferation and differentiation through targeting RUNX2 and WNT/β-catenin signaling in osteoporotic rats [30]. MiRNA-24-2-5p is also a critical regulator of osteogenesis participating in osteogenic differentiation by targeting and post-transcriptionally regulating expression of transcription factor T-cell factor-1 (TCF-1) in osteoblastic cells, while overexpression of miRNA-24-2-5p significantly inhibits osteogenic differentiation [31] in murine osteoprogenitors cells and bone mesenchymal stem cells.

Our results highlight the presence of an epigenetic molecular component in HIV-associated bone loss that is associated with down-regulation of both osteoclastogenesis (decreased relative expression of miRNA-21-5p) and osteoblastogenesis (increased relative expression of miRNA-24-2-5p). On the other hand, decreased relative expression of miRNA-23a-3p is hypothesized to enhance osteoblast function through releasing RUNX2 transcriptional activity, probably as a feedback regulatory mechanism of bone tissue on HIV-induced suppression of bone turnover.

Bone remodeling status in HIV infection has been studied both in treatment-naive individuals and in the first years after HAART initiation, demonstrating, however, conflicting results [50,51,52], while data are scarce concerning HIV+ with long-term HAART. In our study, bone turnover markers did not differ significantly between HIV+ with osteoporosis and HIV+ with normal bone mass, or between HIV+ and HIV− individuals. However, the significantly altered relative expression of miRNAs in serum in the cohort of HIV+ points to a significantly altered bone remodeling status at the tissue level that may distinguish among HIV+ those with HIV-associated osteoporosis.

We did not find significant differences in the miRNA panel used in this study between HIV+ and HIV− individuals, except for the decreased relative expression of miRNA-124-3p. Expression of miRNA-124 inhibits osteoclastogenesis by suppressing the nuclear factor of activated T-cells 1 (NFATc1) and receptor activator of nuclear factor kappa-B ligand (RANKL)-mediated osteoclast differentiation of mouse bone marrow macrophages [36]. The differential expression of miRNA-124-3p in HIV+ compared to HIV− individuals combined with the significant association of miRNA-124-3p expression with TBS, an index of bone microarchitecture [46], in HIV+ further support an epigenetic effect of HIV infection and HAART on bone metabolism and bone remodeling.

The observation of decreased relative expression of miRNA-23a-3p and miRNA-21-5p in the serum of HIV+/OP+ is in line with our previous results in women with PMO [22] and CKD-MBD [25], suggesting a common expression pattern of circulating miRNAs in patients with osteoporosis independent of the underlying cause (HIV infection, estrogen withdrawal, or CKD). In addition, serum expression of miRNA-21-5p in PMO showed in our previous study a 66% sensitivity and 77% specificity in distinguishing among women with PMO those with a vertebral fracture [22]. This is in accordance with our present results in HIV+/OP+ group, underlying the prognostic value of this particular miRNA in identification of bone disease independent of the underlying pathophysiology.

Our study has several limitations. First, we chose to carry out analysis only in a male population of HIV+, thus, our results should be treated with caution if considered in regard to pre- and postmenopausal women with HIV-associated bone loss. This decision was mainly based on the fact that approximately 80% of HIV+ who were under regular control in our department were males. In addition, since we used the same panel of miRNAs that we had previously shown to be differentially altered in PMO, we needed to eliminate from our results the role of estrogen withdrawal in the bone loss associated with HIV by excluding females from the analysis. Second, the design of our study was cross-sectional and, therefore, we could not address bone remodeling status over time, nor could we assess the effect of HAART on the relative expression of the selected miRNAs linked to bone metabolism. Third, the number of participants was relatively small, mainly because HIV+ individuals with osteoporosis who were currently on anti-osteoporotic treatment or had been treated in the past were excluded from the analysis, further restricting our reference population. Fourth, our analysis was not based on a PCR array method but instead we selected a prespecified panel of miRNAs. Regarding this point, however, we could argue that we chose specific miRNAs that are known to regulate key genes of bone remodeling because our research was focused on epigenetic mechanisms of HIV-associated bone loss. Moreover, this specific panel of miRNAs was previously shown to be altered in other causes of bone loss, such as PMO and CKD-MBD, and, thus, it was intriguing to test whether there is a common pattern of miRNA expression profile in bone loss related to different pathophysiology.

Our study provides preliminary results on an epigenetic molecular component in HIV-associated bone loss and deregulation of both osteoclastogenesis and osteoblastogenesis at the tissue level.

Since HAART has considerably increased the life expectancy of HIV+ individuals, understanding the molecular basis of HIV-associated bone loss is a critical step towards early diagnosis and appropriate management of this high-risk population. Research interest in the role of circulating miRNAs as biomarkers that are stable and easily measured has increased greatly over the last few decades, fueled by the technological advances in the analysis of genomic and transcriptomic pathways. MiRNAs have a unique advantage over the other currently used protein-based biomarkers due to the fact that they reflect the ongoing cascade of events at the tissue level. The discovery and validation of miRNA-based diagnostic biomarkers will undoubtedly pave the way to the development of personalized and individualized medicine for patients with chronic and debilitating diseases such as HIV infection.

In conclusion, in this study we demonstrated the potential value of serum relative expression of 3 miRNAs related to bone metabolism; namely, miRNA-21-5p, miRNA-23a-3p, and miRNA-24-2-5p as novel biomarkers in distinguishing HIV+ individuals with osteoporosis.

## Figures and Tables

**Table 1:** Pre-specified panel of selected miRNAs linked to bone metabolism.

Gene Symbol miScript Primer Assay (Catalog # Qiagen)	Predicted Target-Genes	miRNA Sequence	Predicted Mechanism of Action
hsa-miRNA-21-5p MS00009079	*SPRY1; PDCD4; FASLG*	5′UAGCUUAUCAGACUGAUGUUGA	Increases osteoclastogenesis and is up-regulated during RANKL-induced osteoclastogenesis [26,27,28].
hsa-miRNA-23a-3p MS00031633	*RUNX2; SATB2*	5′AUCACAUUGCCAGGGAUUUCC	Decreases osteoblastogenesis through inhibition of the *RUNX2* gene [29,30].
hsa-miRNA-24-2-5p MS00009205	*TCF-1; CALB1; SATB2*	5′UGCCUACUGAGCUGAAACACAG	Decreases osteogenic differentiation through targeting the expression of transcription factor *TCF-1* in osteoblastic cells [31].
hsa-miRNA-26a-5p MS00029239	*ΤΟΒ1; IGF-1*	5′UUCAAGUAAUCCAGGAUAGGCU	Increases bone formation through repressing TOB1 protein expression, negative regulator of BMP/SMAD signaling pathway [32].
hsa-miRNA-29a-3p MS00003262	*SPARC*	5′UAGCACCAUCUGAAAUCGGUUA	Decreases osteonectin-bone matrix protein-synthesis [33].
hsa-miRNA-33a-5p MS00003304	*SATB2; DKK-1; WIF1; OSTF1;* *β-catenin*	5′GUGCAUUGUAGUUGCAUUGCA	Decreases osteoblastogenesis through targeting SATB2 [34] and modulates Wnt signaling [35].
hsa-miRNA-124-3p MS00006622	*NFATC1; NFATC2*	5′UAAGGCACGCGGUGAAUGCC	Decreases osteoclastogenesis by suppressing the *NFATc1* gene [36].
hsa-miRNA-133a-3p MS00031423	*RUNX2; MEG3*	5′UUUGGUCCCCUUCAACCAGCUG	Decreases osteoblastogenesis by targeting the *RUNX2* gene [37] and modulates the expression of long non-coding RNA MEG3 [38].
Cel-miRNA-39-3p MS00019789	Spike-in control	5′UCACCGGGUGUAAAUCAGCUUG	
hsa-SNORD95-11	miScript PCR control		
hsa-SNORD96A-11	miScript PCR control		
hsa-RNU6-2-1	miScript PCR control		

All selected primers were mature miRNAs. The miScript Universal Primer was used as the reverse primer in qPCR. miScript PCR controls were used to enable normalization of qPCR results in miRNA quantification from human samples using the miScript PCR System (Qiagen GmbH, Hilden, Germany). Hsa, homo sapiens; miRNA, microRNA; SPRY1, sprouty homolog 1, antagonist of FGF signaling; PDCD4, programmed cell death protein 4; FASLG, tumor necrosis factor ligand; RUNX2, runt-related transcription factor-2; SATB2, SATB homeobox 2; T-cell factor-1 (Tcf-1); CALB1, calbindin 1, 28kDa; TOB1, transducer of Erb-2, 1; IGF-1, insulin-like growth factor 1;BMP, bone morphogenetic proteins; SMAD, mothers against decapentaplegic homolog; SPARC, secreted protein, acidic, cysteine-rich (osteonectin); DKK-1, dickkopf-1; WIF1, Wnt signaling pathway inhibitory factor 1; OSTF1, osteoclast-stimulating factor 1; NFATC1, nuclear factor of activated T-cells, cytoplasmic, 1; NFATC2, nuclear factor of activated T-cells, cytoplasmic, 2; MEG3, maternally expressed gene 3; Cel, Caenorhabditis elegans.

**Table 2:** Anthropometric and clinical characteristics and biochemical values of the study cohort.

Parameters	HIV+/OP+ (Cases, *n* = 30)	HIV+/OP− (Controls, *n* = 30)	HIV− (*n* = 30)	*p* Value
**Age (years)**	54.7 ± 8.9	52.6 ± 6.0	54.7 ± 5.4	NS
**BMI (kg/m^2^)**	27.5 ± 2.5	23.5 ± 3.7	25.5 ± 1.53	NS
**Smoking,** ***n* (%)**				NS
**Duration of HAART (yrs)**	11.1 ± 6.6	12.6 ± 5.4	ΝΑ	NS
**Duration of treatment with NRTIs (yrs)**	10.9 ± 6.2	11.7 ± 5.4	ΝΑ	NS
**Duration of treatment with TDF (yrs)**	6.6 ± 4.5	5.1 ± 3.2	ΝΑ	NS
**Duration of treatment with NNRTIs (yrs)**	2.9 ± 4.5	4.9 ± 5.5	ΝΑ	NS
**Duration of treatment with PIs (yrs)**	6.7 ± 4.8	5.4 ± 5.2	ΝΑ	NS
**Duration of treatment with INs (yrs)**	1.2 ± 2.0	1.2 ± 1.9	ΝΑ	NS
**LS T-score**	−1.78 ± 1.1	0.67 ± 1.4	−0.35 ± 1.13	a
**LS-BMD (g/m^2^)**	1.007 ± 0.14	1.305 ± 0.17	1.177 ± 0.13	a
**TBS**	1.24 ± 0.12	1.279 ± 0.13	1.291 ± 0.15	a
**LFN T-score**	−1.81 ± 1.0	0.14 ± 0.85	−0.68 ± 0.78	a
**LFN BMD (g/m^2^)**	0.810 ± 0.08	1.05 ± 0.10	1.000 ± 0.11	a
**LH T-score**	−1.71 ± 0.69	0.26 ± 0.77	−0.61 ± 0.81	a
**LH BMD (g/m^2^)**	0.892 ± 0.16	1.152 ± 0.14	1.013 ± 0.11	a
**RFN T-score**	−2.01 ± 0.62	−0.48 ± 0.6	−0.38 ± 0.93	a
**RFN BMD (g/m^2^)**	0.804 ± 0.07	1.059 ± 0.09	0.962 ± 0.09	a
**RH T-score**	−1.77 ± 0.64	0.14 ± 0.65	−1.00 ± 0.93	a
**RH BMD (g/m^2^)**	1.053 ± 0.84	1.119 ± 0.09	0.939 ± 0.12	a
**Serum creatinine** **(NR: 0.5–1.2 mg/dL)**	1.0 ± 0.2	0.9 ± 0.1	1.0 ± 0.1	NS
**Serum calcium #** **(NR:8.2–10.6 mg/dL)**	9.2 ± 0.43	9.3 ± 0.4	9.2 ± 0.23	NS
**Serum phosphate** **(NR:2.7–4.5 mg/dL)**	2.9 ± 0.59	2.9 ± 0.6	3.2 ± 0.45	NS
**Intact PTH** **(NR:10–65 pg/mL)**	36.7 ± 12.56	44.2 ± 10.19	42.7 ± 15.1	NS
**Serum 25-OH-vitamin D (ng/mL)**	16.45 ± 7.66	16.5 ± 7.1	23.9 ± 16.3	NS
**Serum P1NP (ng/mL)**	43.8 ± 12.4	46.9 ± 11.9	35.7 ± 9.3	NS
**Serum β-CTX (ng/L)**	320 ± 119.2	330 ± 95.8	288 ± 116.1	NS
**Clinical/morphometric vertebral fractures**	0	0	0	NA

a: *p* < 0.05 comparisons performed within the HIV+ cohort (HIV+/OP+ vs. HIV+/OP−). NS: not-significant (*p* > 0.05) comparisons were performed between the 3 groups of HIV+/OP+, HIV+/OP− and HIV-controls (ANOVA). HIV+/OP+, individuals with HIV infection and osteoporosis/osteopenia; HIV+/OP−, individuals with HIV infection and normal bone mass; HIV−, individuals without HIV infection; yrs, years; BMI, body mass index; HAART, highly active antiretroviral therapy; NRTIs, nucleoside analog reverse-transcriptase inhibitors; TDF, tenoforvir; NNRTIs, non-nucleoside analog reverse-transcriptase inhibitors; PIs, protease inhibitors; Ins, integrase inhibitors; LS, lumbar spine; BMD, bone mineral density; TBS, trabecular bone score; LFN, left femoral neck; LH, left total hip; RFN, right femoral neck; RH, right total hip; NR, normal range; NA, not applicable.

## Data Availability

Not applicable.
